# Associations of sport participation with self-perception, exercise self-efficacy and quality of life among children and adolescents with a physical disability or chronic disease—a cross-sectional study

**DOI:** 10.1186/s40798-018-0152-1

**Published:** 2018-08-15

**Authors:** Saskia J. te Velde, Kristel Lankhorst, Maremka Zwinkels, Olaf Verschuren, Tim Takken, Janke de Groot, on behalf of the HAYS study group, F. J. G. Backx, J. F. de Groot, K. M. Lankhorst, T. C. W. Nijboer, T. Takken, D. W. Smits, O. W. Verschuren, J. M. A. Visser-Meily, M. J. Volman, H. W. Wittink

**Affiliations:** 1 University of Applied Sciences, Utrecht, the Netherlands; 2 Center of Excellence for Rehabilitation Medicine, Brain Center Rudolf Magnus, University Medical Center Utrecht and De Hoogstraat Rehabilitation, Utrecht, the Netherlands; 3 Child Development and Exercise Center, University Medical Center Utrecht, Utrecht, the Netherlands; 4 Netherlands Institute for Health Services Research, Utrecht, The Netherlands

**Keywords:** Sport, Disability, Psychosocial, Quality of life

## Abstract

**Background:**

Little evidence is available about how sports participation influences psychosocial health and quality of life in children and adolescents with a disability or chronic disease. Therefore, the aim of the current study is to assess the association of sports participation with psychosocial health and with quality of life, among children and adolescents with a disability.

**Methods:**

In a cross-sectional study, 195 children and adolescents with physical disabilities or chronic diseases (11% cardiovascular, 5% pulmonary, 8% metabolic, 8% musculoskeletal/orthopaedic, 52% neuromuscular and 9% immunological diseases and 1% with cancer), aged 10–19 years, completed questionnaires to assess sports participation, health-related quality of life (DCGM-37), self-perceptions and global self-worth (SPPC or SPPA) and exercise self-efficacy.

**Results:**

Regression analyses showed that those who reported to participate in sports at least twice a week had more beneficial scores on the various indicators compared to their peers who did not participate in sport or less than twice a week. Those participating in sports scored better on all scales of the DCGM-37 scale, on the scales for feelings of athletic competence and children but not adolescents participating in sports reported greater social acceptance. Finally, we found a strong association between sport participation and exercise self-efficacy.

**Conclusions:**

This study provides the first indications that participating in sports is beneficial for psychosocial health among children and adolescents with a disability. However, more insight is needed in the direction of the relationships.

## Key points

- Children and adolescents with a physical disability or chronic disease who are participating in sports scored better on all scales of the DCGM-37 scale reflecting a better health-related quality of life
- Participating in sports was associated with feelings of athletic competence in children and adolescents with a disability or chronic disease. In addition, children, but not adolescents, participating in sports reported higher feelings of social acceptance.
- Sport participation among children and adolescents with a physical disability or chronic disease was strongly positively associated with exercise self-efficacy.

## Background

Physical activity and sports participation have several health benefits for people of all ages [1]. Engaging in sufficient levels of physical activity improves cardiopulmonary health, strength, flexibility and endurance, and has been related to reduce risks for cardiovascular diseases and specific cancers [2, 3]. Moreover, physical activity, especially sports, exercise and leisure time activities, has been related to reductions in mortality [4]. In addition, due to its social nature, sports participation provides opportunities for social interaction, companionship and may therefore have greater benefits for social and mental well-being than other domains of physical activity [5–8]. Furthermore, sports participation may enhance health-related quality of life in adults as well as in children and adolescents [9, 10]. Health-related quality of life is a broadly defined construct evaluating the health status from the person’s perspective covering physical, emotional, mental, social and functional domains [11] and has been used in evaluations of sport and exercise interventions (e.g. [12, 13]).

The benefits of sport and physical activity for psychosocial health, i.e. one’s mental processes, self-reflections and interactions with others, are universal and not restricted to healthy adults and typically developing children and adolescents. In the contrary, children and adolescents with a chronic disease or physical disability (CDPD) may benefit even more [14]. Both children with a physical disability as well as children with a chronic disease may experience similar barriers for sport participation. They both have lower fitness levels, lower levels of physical activity and a higher prevalence of overweight and obesity [15–17]. Sport and physical activity does not only improve the physical functioning and physical independence in both groups, but also it may enhance inclusion and well-being. For example, a review about leisure time activities and quality of life among children and adolescents with neurological disabilities reported that participation in active leisure time activities was associated with better physical well-being, improved sense of self, emotional well-being and promote social well-being [18]. Unfortunately, children and adolescents with CDPD engage less often in physical activities and sports [17, 19, 20]. Furthermore, these children and adolescents may experience low levels of self-worth and quality of life due to their physical limitations and body image concerns [21–24]. Therefore, insight in the potential beneficial effects of physical activity and in particular sports participation, as a specific sub-domain of physical activity, is important for subsequent interventions promoting sports participation among children and adolescents with disabilities.

So far, only a few studies addressed the association of sports participation with psychosocial health and quality of life in children and adolescents in general or in children and adolescents with CDPD. Dinomais and colleagues [25] showed that young people with disabilities who participated in competitive sports scored high on social functioning, which is in line with the review by Sahlin and Lexel [9] who concluded that children and adolescents with a disability who engaged in sports reported similar scores on self-concept than non-disabled athletes. However, it remains unclear whether positive associations were the result of the physical activity itself, or of the social interaction and learning environment of the sports club. A study among 15–69 year-old French men and women showed that across all levels of physical activity, sports participation was positively associated with quality of life [26]. In addition, the review by Eime and colleagues [5] reported that club- and team-based sports participation resulted in better psychosocial health outcomes than individual forms of sports.

The aforementioned studies used different indicators, each measuring different but related aspects of psychosocial health. Positive self-worth, self-perceptions, self-esteem, social support and self-efficacy are acknowledged indicators of psychosocial health and quality of life [23, 27]. Involvement in sports, and the interaction with others in this context, allows children and adolescents to develop their self-concept, especially related to the physical and social domains [28]. A systematic review found that physical activity was strongly associated with perceived (athletic) competence [29]. A meta-analysis supported the proposition that physical activity positively influences body image, and this effect was larger in adolescents compared to students, adults and older adults [30]. In summary, participation in physical activity may have an impact on many aspects of psychosocial health; however, the evidence available about how sports participation influences psychosocial health and quality of life in children and adolescents with a disability is still scarce. Therefore, the aim of the current study is to assess the association of sports participation with psychosocial health, specifically, self-perceptions, exercise self-efficacy and quality of life, among children and adolescents with a disability. Based on previous studies, we hypothesise that children and adolescents who participate in sport have more favourable scores on these indicators of psychosocial health and quality of life.

## Methods

### Design and sample

Cross-sectional data from two related studies using identical outcomes were combined. Firstly, the cross sectional Health in Adapted Youth Sports (HAYS) study that includes children and adolescents aged 10–19 years old with a chronic disease or physical disability (CDPD) [31]. Secondly, the baseline data from the Sports-2-Stay Fit (S2SF) study which is a clinically controlled trial among children and adolescents aged 6–19 years with a CDPD [32].

For both studies, children and adolescents were recruited through patient organisations, paediatric physical therapy practices, Wilhelmina Children’s Hospital in Utrecht, De Hoogstraat Rehabilitation Center, Fitkids and schools (for special education) in the Netherlands.

Inclusion criteria were having a physical disability or a chronic disease (cardiovascular, pulmonary, musculoskeletal or neuromuscular disorder), aged between 10 and 19 years (HAYS) or between 6 and 19 years (S2SF), ability to understand simple instructions, able to perform physical fitness tests. Children and adolescents were not eligible for participation in these studies if they had a progressive disease, used an electric wheelchair, participated in other (research) projects that may influence the results of the current studies, or did not sign the informed consent form.

For the current study, children and adolescents were included in the analyses if they had valid data on sports participation and valid data on at least one of the outcome variables (quality of life, self-efficacy, self-perceptions, general self-worth). Eight children and adolescents from the S2SF-study did not complete the online questionnaires during their first assessment, but did during the second assessment (8 weeks later). For that reason, we included data from these eight children or adolescents from their second assessment. We assumed that even though their scores may have been improved in that 8-week period, this will not affect the association between sports participation and these variables.

In total, 197 participants had complete data on sports participation, of whom two had no valid data on the outcome variables. Of the remaining 195 participants, 177 participants had complete data on all outcome variables, another 13 participants had valid data on two of the three outcome variables and 5 participants had valid data on only one outcome variables. The distribution of valid data on the outcome variables did not differ by sport participation status (*p* = 0.428) or by diagnosis (ACSM category) (*p* = 0.346) or by gender (*p* = 0.393). We included 195 children and adolescents who had complete data on sports participation and at least one outcome variable. Of those included children and adolescents, 145 participated in the HAYS study, while 50 participated in the S2SF study.

### Procedures

The procedures and protocols for the HAYS and the S2SF study have been described elsewhere in more detail [31, 32]. Briefly, participants who agreed to participate and met the inclusion criteria were scheduled for an assessment at the lab (University of Applied Science, Utrecht). 1 week before this visit to the laboratory, the participants or their parents received a secured link to online questionnaires assessing exercise self-efficacy and quality of life. These questionnaires took about 10–15 min to complete. When the children visited the laboratory, they were first asked to complete the questionnaires on self-perceptions and global self-worth in the presence of one of the researchers or research assistants, which took about 10 min. When they finished the questionnaires their physical fitness, cognition and cardiovascular health was assessed, which took on average about 2 h [31, 32].

The studies were approved by the Medical Ethics Committee of the University Medical Center Utrecht, the Netherlands. (METC number: 14-332/c and 14-118/m). All participants and the parents of participants under 18 years of age provided their informed written consent. Studies were conducted in accordance with the Helsinki Declaration.

### Measurements

#### Independent variable

##### Sports participation

Sports participation was assessed by means of a questionnaire that was completed before the start of the physical fitness tests. Sports participation was identified using three standardised questions used by the National Institute for Public Health and the Environment (RIVM) [33] (1) “do you participate in organized sports?” (2) “what is/are the type of organized sport(s)?” and (3) “what is the frequency of participation in organized sports per week?”. When participants indicated that they participate in organised sports at least two times per week, they were classified as ‘participating in sports’, all others were classified as ‘non-sporting’. This cut-off was based on the Consensus statement for the Dutch Guidelines for Physical Activity for youth (< 18 years old) [34]. This guideline was developed for typically developing children and was in place at the time of the start of the current research project. It advises that children and adolescents should be physically active for at least 1 h per day at at least a moderate intensity. In addition, children and adolescents should engage in activities that specifically address physical fitness at least two times a week. Sport activities are typically activities in which physical fitness, including strength, flexibility and coordination, are addressed. However, there are no universally accepted guidelines for children and adolescents with a chronic disease of physical disability and Van Brussel and colleagues [35] advise to use a training frequency of minimally two times per week. This is in line with recommendations for people with cerebral palsy that were based on a comprehensive literature review, expert opinions and extensive clinical experience [36].

#### Dependent variables

##### Health-related quality of life

To evaluate the quality of life, the Dutch version of the Disabkids (DCGM-37) was used, which was completed online by either the participant alone (*n* = 45), together with one of the parents/caregivers (*n* = 54) or the parent alone (*n* = 9) (unknown for *n* = 89). This questionnaire measures the quality of life and the independence of children and adolescents with chronic health conditions. The questionnaire has been used in other paediatric populations and has been tested for internal consistency and validity [37–39] The questionnaire includes 37 items that cover six subscales, i.e. mental independence, mental emotion (inner strength), social inclusion, social exclusion (social equality), physical limitations (physical ability) and physical treatment (i.e. the impact of taking medication, receiving injections, etc.) [38]. All items were scored on a 5-point Likert scale ranging from 1 = never to 5 = always. For each scale, a sum score was calculated by following the instructions of the developers of the DCGM-37 and the provided syntax file [38]. If one item was missing, this missing value was substituted by the mean of the non-missing values. If more than 1 item of a domain was missing, no sum score was calculated. These scores were transformed so that they had a range from 0 to 100 with higher scores indicating a higher perceived quality of life.

##### Self-perception and global self-worth

To evaluate self-perception, the Dutch translations of the self-perception profile for children (SPPC) and for adolescents (SPPA) were used [40]. We used the children’s version for children of 12 years old and younger, or with cognitive capabilities of this age group, and the adolescent version for those older than 12 years. Based on the researcher’s expertise, an older child was provided the children’s version or when the child clearly was not able to complete the adolescent version or did not understand the questions. The children’s version uses 36 items to measure five domains, i.e. ‘scholastic competence’, ‘social acceptance’, ‘athletic competence’, ‘physical appearance’, ‘Behavioural conduct’, and the general concept ‘global self-worth’ [41, 42]. All six scales consist of 6 items that include bipolar statements (e.g. ‘some kids feel they are very good at school, but other kids worry about whether they do the schoolwork assigned to them’). The children have to choose which of the two statements resembles them, and how much, i.e. ‘sort of true for me’ or ‘really true for me’. All items were scored on a 4-point scale and sum scores were calculated. The questionnaire has been tested in a Dutch norm group and showed reasonable to good internal consistency and test-retest reliability [42].

The adolescent version includes 35 items which make up 7 scales, i.e. ‘scholastic competence’, ‘social acceptance’, ‘athletic competence’, ‘physical appearance’, ‘behavioural conduct’, ‘close friendship’ and the general concept ‘global self-worth’. Similar to the child version of the questionnaire, all items included bipolar statements that were scored on a 4-point scale by which the adolescents indicated which statement resembled them ‘sort of true’ or ‘really true’ [43].

If one item of the scale was missing, the scale score was not calculated (*n* = 3). All scores on each scale were compared to Dutch norm scores to indicate whether the participants scored ‘below average’, i.e. below the 15th percentile, or above average, i.e. at or higher than the 85th percentile. For the child version, different norms have been formulated for boys and girls [41]. For the adolescent version, different norms were formulated by school level for the scales ‘scholastic competence’ and ‘behavioural conduct’ and a distinction by sex was made for all scales except ‘social acceptance’ [43]. In the current study, results regarding these norm scores were only used for descriptive purposes.

##### Exercise self-efficacy

To assess whether sport participation was associated with exercise self-efficacy, the Dutch version of the Exercise Self-Efficacy Scale [44, 45] was filled out digitally at home by the child. Self-efficacy is a well-known behavioural determinant and described in several behavioural theories such as the Social Cognitive Theory by Bandura [46] in which self-efficacy is seen as important influencer of behaviour. In the current study, we hypothesise that behaviour, in this study engaging in sport, can have a positive impact on self-efficacy in relation to physical activity and exercise. This hypothesis is based on the fact that mastery experiences and vicarious experiences are important sources of self-efficacy [47] and when participating in sport, children may learn new skills and may see others like themselves performing specific tasks or behaviours. The questionnaire takes approximately 2 min to complete. The questionnaire consists of 10 items about the level of self-confidence with regard to performing regular physical activities and exercise that could be rated on a 4-point Likert scale ranging from ‘not true at all’ to ‘always’. A sample item is “I am confident that I can overcome barriers and challenges with regard to physical activity and exercise if I try hard enough”. A sum score was calculated when all 10 items were answered. Internal consistency of the scale was high, Cronbach’s alpha = 0.99. A higher score indicates a higher exercise self-efficacy. Validity and reliability of the scale have been tested in a sample with spinal cord injury and showed good validity and adequate reliability [44].

#### Potential confounders and co-variates

##### Demographic variables

The demographic questionnaire that included questions about date of birth, sex and school level.

##### Medical diagnosis

The general questionnaire assessed the medical diagnosis. The medical diagnoses were further classified in categories according to The American College of Sports Medicine (ACSM) [48]: 1. Cardiovascular (e.g. ventricular septal defect, tetralogy of Fallot, cardiomyopathy), 2. Pulmonary (asthma), 3. Metabolic (diabetes), 4. Musculoskeletal or orthopaedic (amputation, clubfoot, hereditary multiple exostoses-multiple osteochondromas, congenital anomalies), 5. Neuromuscular (cerebral palsy, spina bifida, neurofibromatosis, Kabubi syndrome, centronuclear myopathy, psychomotor retardation, Martsolf syndrome, acquired brain injury), 6. Immunological or haematological (rheumatism, Fanconi anaemia), 7. Cancer (tumour in hypophysis), 8. Epilepsy. In the regression analyses, categories were merged distinguishing disabilities or disease related to the metabolic system or oxygen transport (1. Cardiovascular, 2 Pulmonary, and 3. Metabolic) from the other disabilities or diseases, mostly related to the musculoskeletal or neurological system.

##### School type

Children participating in the current study attended either regular education or special education for children with physical disabilities (so called mytyl schools). These schools, dedicated to children with CDPD, have similar learning objectives as regular schools, but the children receive additional attention and support. Children attending special education may have different references for social comparison which may influence how they perceive themselves. Therefore, school type was added as a potential confounder in the analyses.

#### Statistical analyses

Continuous data was described by means or medians and standard deviations or interquartile ranges and categorical data by frequencies and proportions. Crude comparisons on the outcome variables between the sporting and non-sporting participants were made using ANOVA. Adjusted associations between sports participation and the outcome variables were estimated by linear (or logistic) regression analyses. Assumptions were checked, and residuals showed an acceptable normal distribution, except for the Exercise Self-Efficacy scale. Therefore, the scale was dichotomized at the third tertile (score ≥ 37) and associations with sports participation were assessed by means of logistic regression analyses. Sensitivity analyses were run with and without outliers, i.e. those with standardised residual scores below − 3 or higher than 3.

Different models will be presented, unadjusted as well as adjusted for potential confounders’ sex, age, school type, diagnosis or ACSM category. All analyses were checked for potential interaction by age. In case of significant interactions, results will be presented for two age groups. For the analyses, the ACSM categories were merged into two categories, i.e. one including those with cardiovascular, pulmonary and metabolic conditions, and another including those with musculoskeletal, neuromuscular, immunological, cancer or epileptic conditions. Results are reported as regression coefficients or odds rations and their 95% confidence intervals.

## Results

Of the 195 children included in the current study, 96 were classified as participating in sports (see Table 1). Based on the ACSM classification, most children (*n* = 104 (52.8%)) had a neuromuscular condition (Table 1), followed by cardiovascular disease (*n* = 22 (11.3%)). One third of the children attended special education.

**Table 1:** Characteristics of the study sample (*N* = 195)

	Mean	Standard deviation
Age (years)	14.3	2.8
	n	%
Study^1^ (HAYS)	145	74.4
Sex (boys)	116	59.5
Sport participation^2^ (yes)	96	49.2
ACSM		
Cardiovascular	22	11.3
Pulmonary	9	4.6
Metabolic	16	8.2
Musculoskeletal or orthopaedic	16	8.2
Neuromuscular	103	52.8
Immunological or haematological	18	9.2
Cancer	3	1.5
Epileptic	8	4.1
Education type^3^		
Special-education	65	33.3

^1^Participating in either the HAYS or the S2SF study

^2^Sport participation is defined here as participating in sport at least two times a week

^3^Education type could be regular education or special education

Table 2 shows the descriptive statistics regarding the outcome variables. The mean scores on the different subscales for the DCGM-37 varied, it was lowest for the social exclusion scale (62.9 (17.7)) and highest for the mental emotion (77.9 (17.8)) and physical treatment (77.1 (22.1)). When looking at the norm scores for the SPPC and SPPA, a relatively high proportion of children scores below the norm for social acceptance (SPPA, 21.3%) and athletic competence (SPPC, 25%; SPPA, 23.45). On the other hand, relatively many children score above the 85th percentile for scholastic competence (SPPA, 29.2%), social acceptance (SPPC, 25.7%), athletic competence (SPPC, 23.6%) and behavioural conduct (SPPC, 35.0%; SPPA, 33.3%).

**Table 2:** Descriptive statistics for quality of life, self-perceptions, global self-worth and self-efficacy

DISABKIDS (DCGM-37)^1^	*N*	Mean	SD	Median	p_25_	p_75_	Min	Max
Mental independence (range 0–100)	187	75.8	14.6	79.2	66.7	87.5	33.3	100
Mental emotion (range 0–100)	187	77.9	17.8	78.6	67.9	92.9	28.6	100
Social inclusion (range 0–100)	187	62.9	17.7	66.7	50.0	75.0	16.7	100
Social exclusion (range 0–100)	187	75.8	17.9	79.2	66.7	91.7	12.5	100
Physical limitations (range 0–100)	187	68.0	18.5	70.8	54.2	83.3	12.5	100.0
Physical treatment (range 0–100)	87	77.1	22.1	83.3	66.7	91.7	20.8	100
General (31 items) (range 0–100)	187	72.3	1.5	72.6	61.3	83.9	29.0	100
Self-perception profile for children (SPPC)	*N*	Mean	SD	Median	p_25_	p_75_	% <p_15_^2^	% > p_85_^3^
Scholastic competence (6 items, range 6–24)	140	17.1	4.0	17	14	20	16.4	18.6
Social acceptance (6 items, range 6–24)	140	18.2	4.2	18	16	22	15.0	25.7
Athletic competence (6 items, range 6–24)	140	17.6	4.4	18	15	21	25.0	23.6
Physical appearance (6 items, range 6–24)	140	19.3	4.4	20	17	23	15.7	19.3
Behavioural conduct (6 items, range 6–24)	140	18.8	3.8	19	17	22	11.4	35.0
Global self-worth (6 items, range 6–24)	139	20.2	3.3	21	18	23	13.7	22.3
Self-perception profile for adolescents (SPPA)	*N*	Mean	SD	Median	p_25_	p_75_	% < p_15_	% > p_85_
Scholastic competence (5 items, range 5–20)	48	14.8	2.9	15	13	17	14.6	29.2
Social acceptance (5 items, range 5–20)	47	14.8	2.9	15	13	17	21.3	6.4
Athletic competence (5 items, range 5–20)	47	13.1	4.0	14	10	16	23.4	14.9
Physical appearance (5 items, range 5–20)	47	14.0	3.2	14	12	16	12.8	10.6
Behavioural conduct (5 items, range 5–20)	48	15.9	2.9	16	15	18	4.2	33.3
Close friendship (5 items, range 5–20)	47	17.6	2.2	18	17	19	4.2	10.4
Global self-worth (5 items, range 5–20)	46	15.7	2.7	16	14	18	10.9	10.9
	N	Mean	SD	Median	p_25_	p_75_	Min	Max
Exercise self-efficacy (sum score 10 items)	187	33.8	5.3	35	31	38	14	40
		n	%					
Exercise self-efficacy (dichotomized, > third tertile)	187	71.0	38.0					

^1^All subscales are scored from 0 to 100 with higher scores indicating higher self-perceived health-related quality of life; p_25_—25th percentile; p_75_—75th percentile

^2^<p_15_ based on a reference sample

^3^ > p_85_ base on a reference sample

Table 3 displays the unadjusted comparisons on the outcome variables between sporting and non-sporting participants. Those participating in sports scored higher on all subscales of the DCGM-37, except on the physical treatment scale. The significant differences in the unadjusted analyses remained statistically significant after adjustment for potential confounders, indicating that school type and medical diagnosis did not substantially confound the association (Table 4).

**Table 3:** Unadjusted comparisons on quality of life, self-perceptions, global self-worth and self-efficacy between sporting and non-sporting participants

	Sporting				Non-sporting				*P* value
	*N*	Mean	SD	Median	*N*	Mean	SD	Median	
DISABKIDS (DCGM-37)^1^									
Mental independence (range 0–100)	93	79.2	13.8	79.2	94	72.4	14.6	75.0	*0.001*
Mental emotion (range 0–100)	93	81.9	16.7	85.7	94	73.9	18.1	75.0	*0.002*
Social inclusion (range 0–100)	93	68.6	17.5	70.8	94	57.2	16.0	58.3	*< 0.001*
Social exclusion (range 0–100)	93	79.9	15.9	83.3	94	71.8	18.8	75.0	*0.002*
Physical limitations (range 0–100)	93	72.0	18.0	70.8	94	64.1	18.3	62.5	*0.003*
Physical treatment (range 0–100)	48	78.6	20.0	83.3	39	75.2	24.6	83.3	0.474
General (31 items) (range 0–100)	93	76.5	13.9	79.8	94	68.1	14.0	69.4	*< 0.001*
Self-perception profile for children (SPPC)									
Scholastic competence (6 items, range 6–24)	66	17.0	4.0	17.0	74	17.3	3.9	18.0	0.639
Social acceptance (6 items, range 6–24)	66	19.3	3.3	19.0	74	17.2	4.7	18.0	*0.003*
Athletic competence (6 items, range 6–24)	66	19.1	3.9	20.0	74	16.2	4.5	17.0	*< 0.001*
Physical appearance (6 items, range 6–24)	66	19.5	4.0	20.0	74	19.1	4.7	20.0	0.601
Behavioural conduct (6 items, range 6–24)	66	18.8	3.6	19.0	74	18.8	3.9	19.0	0.989
Global self-worth (6 items, range 6–24)	66	20.3	3.3	21.0	73	20.0	3.2	21.0	0.582
Self-perception profile for adolescents (SPPA)									
Scholastic competence (5 items, range 5–20)	27	15.1	2.6	16	21	14.4	3.3	15	0.453
Social acceptance (5 items, range 5–20)	26	14.7	3.5	16	21	14.8	2.0	15	0.928
Athletic competence (5 items, range 5–20)	27	14.3	3.2	14	20	11.6	4.5	11.5	*0.022*
Physical appearance (5 items, range 5–20)	27	14.3	2.8	15	20	13.5	3.8	13.5	0.409
Behavioural conduct (5 items, range 5–20)	27	15.8	3.0	16	21	16	2.8	16	0.749
Close friendship (5 items, range 5–20)	28	17.8	2.1	18	20	17.4	2.5	17.5	0.560
Global self-worth (5 items, range 5–20)	25	16.0	2.4	17	21	15.4	3.1	15	0.485
Exercise self-efficacy (sum score 10 items)	93	35.2	4.3	36	94	32.4	5.8	33.5	*< 0.001*
		n	%			n	%		
Exercise self-efficacy (dichotomized, third tertile)	93	46	49.5		94	25	26.6		*0.002* ^2^

Italicize data indicate statistical significance

^1^All subscales are scored from 0 to 100 with higher scores indicating higher self-perceived health-related quality of life

^2^Tested with a chi-square test

**Table 4:** Results from regression analyses comparing sporting (1) and non−sporting participants (0) on health−related quality of life, self−perceptions, global self−worth and exercise self−efficacy

	Model 1^a^		Model 2^b^		Model 3^c^	
	*b*	95% CI	*b*	95% CI	*b*	95% CI
DISABKIDS (DCGM−37)						
Mental independence (range 0–100)	*6.06*	(*1.83; 10.3*)	*4.68*	(*0.18; 9.18*)	*5.75*	(*1.41; 10.1*)
Mental emotion (range 0–100)	*6.32*	(*1.19; 11.5*)	*6.24*	(*0.74; 11.7*)	*7.06*	(*1.60; 12.5*)
Social inclusion (range 0–100)	*11.0*	(*5.99; 16.0*)	*7.76*	(*2.56; 13.0*)	*8.86*	(*3.80; 13.9*)
Social inclusion (range 0–100)	*7.56*	*(2.35; 12.8*)	*5.63*	(*0.11; 11.1*)	*6.79*	(*1.42; 12.2*)
Physical limitations (range 0 − 100)	*6.75*	(*1.35; 12.1*)	*5.82*	(*0.05; 11.6*)	*7.02*	(*1.39; 12.6*)
Physical limitations (range 0 − 100)	n.a.		n.a.		n.a.	
General (31 items) (range 0 − 100)	*7.50*	(*3.35; 11.7*)	*6.03*	(*1.63; 10.4*)	*7.09*	(*2.86; 11.3*)
Self−perception profile for children (SPPC)						
Scholastic competence (6 items, range 6–24)	− 0.86	(−2.17; 0.45	− 0.36	(−1.76; 1.03	− 0.42	(− 1.81; 0.96
Social acceptance (6 items, range 6–24)	*1.63*	(0.26; 3.01)	*2.00*	(0.52; 3.48)	*2.01*	(0.52; 3.50)
Athletic competence (6 items, range 6–24)	*1.90*	(0.49; 3.31)	*2.69*	(1.21; 4.17)	*2.65*	(1.17; 4.12)
Physical appearance (6 items, range 6–24)	− 0.50	(− 1.95; 0.94)	− 0.23	(− 1.79; 1.33)	− 0.26	(− 1.82; 1.31
Behavioural conduct (6 items, range 6–24)	0.25	(− 0.87; 1.38)	0.48	(− 0.74; 1.69)	0.48	(− 0.74; 1.70)
Global self-worth (6 items, range 6–24)	0.15	(− 0.93; 1.23)	0.50	(− 0.65; 1.66)	0.47	(− 0.68; 1.63)
Self−perception profile for adolescents (SPPA)						
Scholastic competence (5 items, range 5–20)	0.623	(− 1.249; 2.496)	0.504	(− 1.393; 2.401)	0.849	(− 1.242; 2.940)
Social acceptance (5 items, range 5–20)	0.058	(− 1.856; 1.973	0.112	(− 1.844; 2.067)	1.173	(− 0.925; 3.270)
Athletic competence (5 items, range 5–20)	2.385	(− 0.075; 4.845)	*2.542*	(0.043; 5.041)	*3.319*	(0.647; 5.992)
Physical appearance (5 items, range 5–20)	− 0.013	(− 2.029; 2.004)	− 0.132	(− 2.183; 1.919)	−0.030	(− 2.283; 2.222)
Behavioural conduct (5 items, range 5–20)	− 0.241	(− 1.791; 1.309)	− 0.401	(− 1.946; 1.144)	−0.181	(− 1.881; 1.520)
Close friendship (5 items, range 5–20)	0.849	(− 0.592; 2.289)	0.676	(− 0.755; 2.108)	*1.636*	(0.269; 3.002)
Global self-worth (5 items, range 5–20)	− 0.666	(− 2.545; 1.213)	− 0.832	(− 2.723; 1.059)	−0.360	(− 2.473; 1.753)
	OR	95% CI	OR	95% CI	OR	95% CI
Exercise self−efficacy (dichotomized, third tertile)	*2.72*	(*1.47; 5.02*)	*2.24*	(*1.17; 4.27*)	*2.55*	(*1.30; 4.99*)

Italicize data indicate statistical significance

^a^Only adjusted for sex and age

^b^Further adjusted for school type

^c^Further adjusted for ACSM classification (recoded into two categories (cardiovascular, pulmonary, metabolic impairments vs musculoskeletal, neuromuscular, immunological, cancer, epileptic impairments/diseases)

*b*, regression coefficient, reflects the difference in score between non−sporting (0) and sporting (1) children; *95%CI*, 95% confidence interval; *n.a.*, not applicable, too few children who completed this scale

Children participating in sports reported higher scores on social acceptance and athletic competence than their non-sporting peers, while no differences were observed for the other scales of the SPPC (Table 3). These differences became slightly stronger after adjustment for potential confounders (Table 4).

The adolescents who participated in sports, scored higher on athletic competence than the non-sporting adolescents (SPPA, Table 3). Moreover, this difference became slightly larger after adjustment of age, gender, school type and ACSM classification (Table 4). In addition, after adjustment for potential confounders, the scores on the scale for close friendships was significantly higher for adolescents participating in sports compared to their non-sporting peers.

Those participating in sports scored more often in the upper tertile on the exercise self-efficacy scale than their non-sporting peers (Tables 3 and 4). After adjustment for school type and medical diagnosis, the odds ratio decreased slightly, but remained significant. Those who participate in sports were 2.55 more likely to score in the upper tertile for exercise self-efficacy, independent of sex, school type or medical diagnosis (Table 4).

## Discussion

The current study aimed to assess the associations between sports participation and psychosocial health among children and adolescents with a disability. In general, those who reported to participate in sports at least twice a week, had more beneficial scores on the various indicators compared to their peers who did not participate in sports or less than twice a week. This was independent of age, sex, school type or medical diagnosis and largely in line with our hypotheses.

Those participating in sports scored better on all scales of the DCGM-37 scale. The effect sizes were quite substantial, for the total score the difference between the groups was about 7 points. Although the fact that the DCGM-37 distinguishes six sub-scales reflecting different concepts, these sub-scales were strongly correlated in the current sample. Therefore, it is not surprising that the groups differed on all sub-scales. Unfortunately, it is difficult to compare these findings with other studies, due to the fact that other instruments to assess quality of life were used, or that other studies did not made comparisons between sporting and non-sporting participants. However, our findings support the hypothesis that participating in organised sports by children and adolescents with a CDPD can contribute to all domains of quality of life and are in line with two studies among adults [49, 50].

Furthermore, our findings regarding the self-perceptions are in line with the existing reviews among adults, children and adolescents [29, 30], and show that participating in organised sports contributes to feelings of athletic competence in children and adolescents with a CDPD. In addition, children, but not adolescents, participating in sports reported higher feelings of social acceptance. It may be that adolescents have more networks besides a sports club that influence their feelings of social acceptance, while children have a more controlled and limited network and that participating in sports with other children therefore has a greater influence on their feelings of social acceptance. On the other hand, the effect estimate was in favour of the sporting adolescents, and it may be that for these analyses not enough participants were included to show a significant difference. In addition, a study among high school students found a positive association between sports participation and self-belief, that included feelings of social acceptance [51]. Lastly, it may be that children who do not feel accepted withdraw from sport participation. That we did not find significant differences between the groups on most of the other self-perception scales, may be due to the fact that our study population scored relatively high on most scales, at least when compared with the 15th and 85th percentile norm values. However, compared to a study sample described by Shapiro and colleagues, the current sample scored lower on the social acceptance, athletic competence and physical appearance scales [52]. That in the current study no associations between sport participation and self-perception concepts were found may be because of other factors than sports participation play a more crucial role in these scales or that different types of sports may have different effects that could not be detected in the current study design.

Finally, despite the fact that the self-efficacy scale was dichotomized which may have led to reduced power, we found a strong association between sports participation and exercise self-efficacy. Self-efficacy is a well-known behavioural determinant of physical activity [53] assuming that exercise self-efficacy influences physical activity. In the current study, we hypothesised another causal pathway, i.e. that sports participation would result in beneficial scores on a range of psychosocial health indicators, including exercise self-efficacy. Sources of influence of self-efficacy are vicarious experiences or modelling and past experiences [46]; therefore, it can be expected that those who participate in sports see others performing sports, and also experience themselves that they are able to perform sports, which both positively impact on their exercise self-efficacy.

An important limitation of the current study is its cross sectional design limiting the conclusions regarding causality. It may as well be that those who experience a better quality of life, or who feel socially accepted and athletically competent, are more likely to participate in organised sports. Therefore, experimental and longitudinal studies are required to study causal or reciprocal relationships. These longitudinal designs would also allow for mediation analyses to study underlying pathways. For instance, in a longitudinal design, we could analyse whether changes in sport participation result in changes in intermediate variables (e.g. self-perception) that in turn result in changes in health-related quality of life. Another limitation is the crude measure of sports participation, which did not include information on duration, quality and type of sports. It may be that team sports have different impact on psychosocial health indicators than individual sports. Furthermore, we did not report whether they participated in regular sports or in adapted forms of sports. The advantage of participation in adapted forms of sports is that children can better focus on their abilities rather than their inabilities [49]. Additionally, the DCGM-37 was in a few cases (*n* = 9) completed by the parent, which may have biased the findings as parents tend to report lower scores than children [37, 54]. However, if this is a systematic bias, it will not affect the association between sport participation and health-related quality of life. Finally, those defined as participating in sports may perform sports at different intensities and frequencies. In conclusion, the group defined as participating in sport is very heterogeneous, and this may have biased the results such that potential associations between sport participation and the outcome may not have been detected. Therefore, future studies should take these aspects into account and may also investigate how the level of sport participation is related to self-perceptions. However, even with the use of this crude measure for sports participation, significant associations with psychosocial health and quality of life were detected.

## Conclusions

Despite some limitations and considering the fact that this is (one of) the first studies addressing this topic in children and adolescents with a CDPD, the current study provides the first indications that participating in sports is beneficial in this population for their psychosocial health. A next step would be to perform experimental and longitudinal studies to see what type of sports, duration and intensities are most promising for improving psychosocial health.

## Abbreviations

ACSM: American College of Sports Medicine
CDPD: Chronic disease or physical disability
DCGM-37: Dutch version of the Disabkids, health-related quality of life questionnaire, 37 items
HAYS: Health in Adapted Youth Sports
S2SF: Sports-2-Stay Fit
SPPA: Self-Perception Profile for Adolescents
SPPC: Self-Perception Profile for Children
